# A Stereo-Vision System for Measuring the Ram Speed of Steam Hammers in an Environment with a Large Field of View and Strong Vibrations

**DOI:** 10.3390/s19050996

**Published:** 2019-02-26

**Authors:** Ran Chen, Zhongwei Li, Kai Zhong, Xingjian Liu, Yonghui Wu, Congjun Wang, Yusheng Shi

**Affiliations:** State Key Laboratory of Material Processing and Die & Mould Technology, Huazhong University of Science and Technology, Wuhan 430074, China; chenran@hust.edu.cn (R.C.); xingjianliu@hust.edu.cn (X.L.); wuyonghui@hust.edu.cn (Y.W.); walden@hust.edu.cn (C.W.); shiyusheng@hust.edu.cn (Y.S.)

**Keywords:** speed measurement, stereo-vision, large field of view, vibration, calibration

## Abstract

The ram speed of a steam hammer is an important parameter that directly affects the forming performance of forgers. This parameter must be monitored regularly in practical applications in industry. Because of the complex and dangerous industrial environment of forging equipment, non-contact measurement methods, such as stereo vision, might be optimal. However, in actual application, the field of view (FOV) required to measure the steam hammer is extremely large, with a value of 2–3 m, and heavy steam hammer, at high-speed, usually causes a strong vibration. These two factors combine to sacrifice the accuracy of measurements, and can even cause the failure of measurements. To solve these issues, a bundle-adjustment-principle-based system calibration method is proposed to realize high-accuracy calibration for a large FOV, which can obtain accurate calibration results when the calibration target is not precisely manufactured. To decrease the influence of strong vibration, a stationary world coordinate system was built, and the external parameters were recalibrated during the entire measurement process. The accuracy and effectiveness of the proposed technique were verified by an experiment to measure the ram speed of a counterblow steam hammer in a die forging device.

## 1. Introduction

The ram speed of a steam hammer reflects the energy of a forging’s deformation and directly affects the forming performance of forging equipment. Thus, it is an important parameter in the forging process. Due to their advantages of long life, high efficiency, and great energy, hammers [1] driven by steam are used in forging, especially for large workpieces. However, the ram speed of the steam hammer varies as the number of its use cycles increases, which may deteriorate its performance in forming workpieces. Therefore, it must be monitored regularly in practical applications. Currently, contact sensors, such as acceleration sensors [2] and inertial sensors [3,4], are widely used for speed measurement. Although these sensors are relatively accurate, they need to be affixed to the object being measured. Considering that the temperature of the steam hammer is very high, reaching around 300 °C, it is difficult to obtain stable and accurate measurement data when using contact speed sensors, which greatly limits their application.

To overcome the impact of high temperatures, non-contact methods are a good choice. There are various non-contact speed measurement systems, such as the Global Positioning System (GPS) [5,6], laser Doppler velocimetry [7], radar velocimetry [8], and stereo-vision techniques [9,10,11,12,13]. However, when GPS is used indoors, the signal can be disturbed by the building, which ultimately affects its measurement accuracy. Devices for laser Doppler velocimetry and radar velocimetry should be installed on the moving line of a steam hammer and facing the measurement surface. This installation requirement is hard to achieve owing to the complex and dangerous environment in and near forging equipment. In addition, non-contact methods require the sensors to remain stationary during the measurement process, but the high speed and weight of a steam hammer usually cause a strong vibration. Thus, it is difficult to keep a non-contact system stationary in the vibrating environment, which often leads to unreliable measurement results for non-contact measurement methods. Despite the diversity of non-contact speed measurement options, stereo-vision techniques have received increasing attention due to their outstanding advantages, such as easy-to-use setup, multipoint measurement and visualization, and wide range of resolution and applicability. However, in actual application, the size of the steam hammer and its travel distance are large, with a value up to 2 m. To measure the ram speed of steam hammer, the measurement FOV of the stereo-vision system should be larger than that size, which can lead to inaccurate measurement results. The main reason for this is that it is difficult to obtain accurate calibration results in a large FOV. Traditional calibration methods [14,15] require a high-precision calibration target with a size similar to that of the range of the measurement FOV. This creates challenges for a large FOV stereo-vision system because fabricating a high-precision large calibration target is difficult and expensive. In addition, strong vibration can cause the speed measurement to fail. First, strong vibration may change the relative pose between the two cameras. Second, the measurement coordinate system moves in a strongly vibrating environment.

To solve these problems, a bundle-adjustment-based system calibration method is proposed to obtain accurate calibration results when the calibration target is not precisely manufactured. The calibration method is divided into calibrating internal parameters and external parameters. To eliminate the influence of strong vibration, the external parameters are recalibrated during the whole measurement process, and the world coordinate system is based on the steam hammer bracket, which is relatively stationary in respect to the steam hammer. Compared with traditional contact measurement techniques, the stereo-vision method has the advantages of compact configuration, ease of use, and capability for non-contact and multipoint deflection measurement. Additionally, in contrast with non-contact methods, the proposed technique offers an outstanding advantage of flexible system configuration and insensitivity to strong vibration due to its stationary measurement coordinate system. The accuracy and effectiveness of the proposed method were verified by experiments that measured the ram speed of a counterblow steam hammer in a die forging device. In addition, this technique can also be used in displacement measurement in a vibrating environment with a large FOV.

This paper is organized as follows: Section 2 introduces the system configuration, the procedures and principles involved in this system, an overview of the stereo-vision method, the system calibration method for a large FOV, the external parameters calibration method of the stereo-vision system, and speed solutions. Section 3 shows the experimental validations, and Section 4 summarizes the study.

## 2. Stereo-Vision System for Measuring Steam Hammer Speed

### 2.1. System Configuration

**Figure 1:**
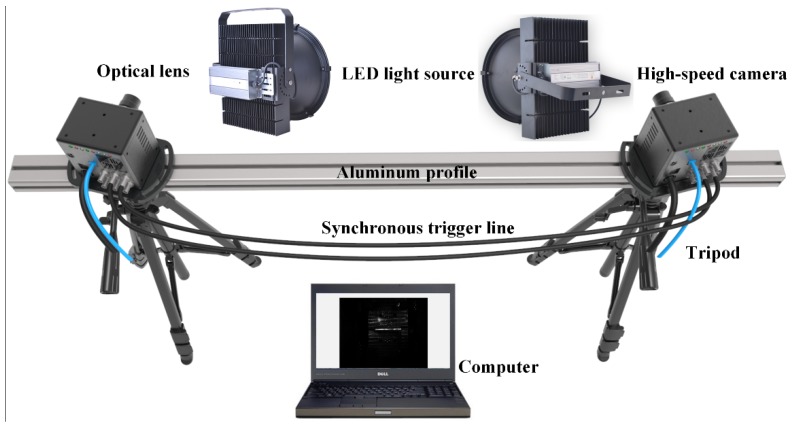
Stereo-vision system for measuring the ram speed of the steam hammer.

The stereo-vision system we developed for measuring the ram speed of a steam hammer is shown in Figure 1. The system consists of two high-speed digital monochrome video cameras (Photron FASTCAM Mini UX100 type 200K-M-16GB, resolution of 1280 × 1024 pixels with 12-bit quantization, maximum frame rate of 4000 frames per second at full resolution, Photron, Tokyo, Japan), two Schneider Xenoplan 1.9/35 lenses with a fixed focal length of 35 mm (Schneider, Bad Kreuznach, Germany), two white LED light sources with a power of 500 W each, an aluminum beam 2 m long, two tripods, and a laptop computer (Dell Precision 4800, Intel^®^ Core i7-4800MQ, 2.7 GHz, 8 GB RAM, Dell, Texas, USA). The video cameras were tightly clamped to the aluminum beam and could be adjusted readily. The two video cameras were synchronized with an internal trigger signal. Images captured by the cameras were stored in the camera’s memory and transmitted to the computer through a gigabit network cable after image acquisition was complete.

It is important to note that the steam hammer has a high ram speed, about 3 m/s, and the duration of impact between the hammer and forging is very short. Thus, to accurately measure the ram speed of the steam hammer, the stereo-vision system uses two high-speed cameras. To ensure the image quality of high-speed cameras with a large FOV, their FOV is illuminated by two high-power LED light sources. To increase the image contrast and signal-to-noise ratio, retro-reflection targets were used which made by mixing glass, silver powder, and resin according to a specific mixing ratio.

### 2.2. Measurement Principles

#### 2.2.1. Stereo-Vision Theory

In the stereo-vision system, the camera can be modeled by a standard pinhole model. An arbitrary 3D point P in the world coordinate system is denoted as $P_{w}$; the ray departing from P and passing through the *ith* camera lens is captured on the camera sensor formed the image point $p_{i}$. In practice, although the lens aberrations distort the shape of the images, the imaging process can be described with a nonlinear camera model [16]:

(1) $$\{\begin{array}{c} s_{i}{{\tilde{p}}^{\prime }}_{i}=A_{i}\left[R_{i}|t_{i}\right]{\tilde{P}}_{w}\\ p_{i}={p^{\prime }}_{i}+\theta (k_{i};{p^{\prime }}_{i}) \end{array},\mathrm{with}A_{i}=\left[\begin{array}{ccc} ax_{i} & 0 & u_{i}\\ 0 & ay_{i} & v_{i}\\ 0 & 0 & 1 \end{array}\right],$$

where $s_{i}$ is a scaling factor, $\tilde{•}$ is the homogenous coordinate, ${•}^{\prime }$ is the ideal image point without distortion; $A_{i}$ is the intrinsic camera matrix which contains the normalized horizontal and vertical direction focal lengths $\left(ax_{i},ay_{i}\right)$ and the principal point coordinates $\left(u_{i},v_{i}\right)$ in the image coordinate system; $\theta (k_{i};•)$ is the lens distortion that parameterized by the distortion coefficients $k_{i}$; and $R_{i}$ and $t_{i}$ are the rotation matrix and translation vector, respectively, which are referred to as extrinsic parameters.

In the binocular vision system, if the corresponding points $p_{1}$ and $p_{2}$ are determined, the 3D coordinates of the homogenous point $P_{w}$ can be reconstructed by a least-squares solution according to Equation (2):

(2) $$\{\begin{array}{c} s_{1}{\tilde{p}}_{1}=A_{1}\left[R_{1}|t_{1}\right]{\tilde{P}}_{w}\\ s_{2}{\tilde{p}}_{2}=A_{2}\left[R_{2}|t_{2}\right]{\tilde{P}}_{w} \end{array},$$

where $s_{1},s_{2},A_{1},A_{2},R_{1},t_{1},R_{2},t_{2}$ can be accurately calibrated before measurement, which will be described in the next Section 2.2.2 and Section 2.2.3.

In the stereo-vision system, the cameras are fixed with respect to each other, the rotation matrix R and translation vector t can be introduced to represent the relative motion between two cameras, which is defined by:

(3) $${\{\begin{array}{c} R=R_{2}R_{1}^{-1}\\ t=t_{2}-R_{2}R_{1}^{-1}t \end{array}}_{1}$$

#### 2.2.2. System Calibration for Large FOV

The essence of stereo-vision system calibration is to solve the internal and external parameters using the known coordinates of multiple 3D points and the corresponding 2D image points. The calibration accuracy directly determines the measurement accuracy of the stereo-vision system. In this paper, a bundle-adjustment-principle-based system calibration method in a large FOV is proposed, and the calibration process is divided into calibrating internal parameters and external parameters.

**Figure 2:**
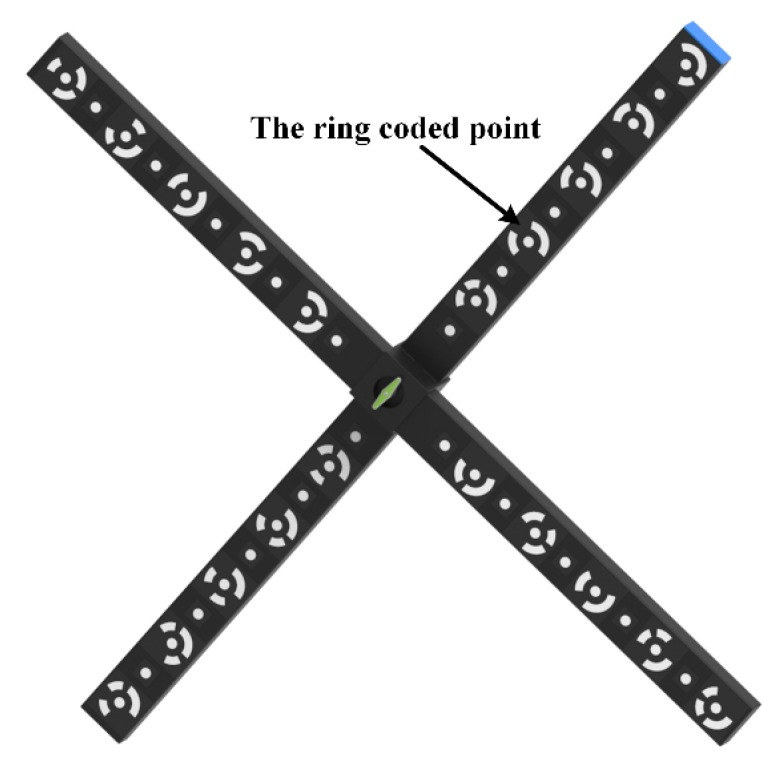
Cross-shaped calibration target.

As shown in Figure 2, a cross-shaped calibration target with ring-coded points and circular points is used. Because the calibration target is portable and easy to manufacture, it is convenient for in-situ calibration, especially for large FOV calibration. The ring-coded points on the calibration target can be identified from an image regardless of their rotation or scale. It is used to correlate the reference points in different images to make the calibration process completely automated. The ring-coded points can be identified by an accurate gray-gradient-based method [17] previously proposed by the authors. In addition, each circular point’s identification number can be easily determined according to the positional relationship between the circular points and ring-coded points.

After identifying the reference points in different images, the corresponding points in these images can be determined. The corresponding points can be also related by epipolar geometry, which can be described by the fundamental matrix F; it contains all the geometric information between two cameras, including the intrinsic parameters and relative rigid motion:

(4) $${{\tilde{p}}^{\prime }}_{2}^{T}F{\tilde{p}}_{1}^{\prime }=0,\mathrm{with}F=A_{2}^{-T}{\left[t\right]}_{x}RA_{1}^{-1},$$

where ${\left[t\right]}_{x}$ is the anti-symmetric matrix by t, and for any vector y, ${\left[t\right]}_{x}y=t\times y$.

To obtain the relative rigid motion, we assume that the cameras are an ideal perspective imaging system that the distortion coefficients $k_{i}$ are 0; thus, we have ${\tilde{p}}^{\prime }=\tilde{p}$, and the fundamental matrix F can be estimated by the classic eight-point algorithm and iterative optimization based on minimizing the distances between reference points and epipolar lines. Furthermore, the initial value of intrinsic parameters in $A_{i}$ can be deduced from the nominal parameters of camera and lens. For example, the principal point coordinates $\left(u_{i},v_{i}\right)$ are assumed to be at the image center. The initial value of the normalized horizontal and vertical focal lengths $\left(ax_{i},ay_{i}\right)$ can be computed by $ax_{i}=f_{i}/dx_{i}$ and $ay_{i}=f_{i}/dy_{i}$, where $f_{i}$ is the nominal focal length, $dx_{i}$ and $dy_{i}$ are the horizontal and vertical pixel size of the camera sensor, respectively. Although the initial value of intrinsic matrix is not very accurate, it is sufficient to gain the essential matrix $E={\left[t\right]}_{x}R$ according to Equation (3). Furthermore, the relative rigid motion of the cameras can be calculated at a low precision level by singular-value-deposition of E [18]. After retrieving the relative rigid motion between cameras, the 3D coordinate of the homogenous control points could be reconstructed by a least-squares solution according to Equation (2).

When calibrating the camera internal parameters, the first image is taken as a reference. Then, the initial 3D coordinates of reference points on the calibration target and the initial external parameters can be solved by the above method. To obtain accurate camera internal parameters and accurate 3D coordinates of reference points, the non-linear least-squares optimization method is used to refine the optical geometry together with the 3D coordinates of reference points. The corresponding cost function is built according to discrepancies between reference points and the expected re-projection data:

(5) $$Cst={\sum_{i=1}^{M}\sum_{j=1}^{N}\left\|p^{ij}-{\hat{p}}^{ij}(A,K,R^{i},t^{i},P_{w}^{j})\right\|}^{2},$$

where superscripts i,j denote the sequence number of images and the reference points respectively; M is the total number of images; N is the total number of reference points; $P_{w}^{j}$ are the *j*th reference points in the world coordinate system; $p^{ij}$ are the image points of *j*th reference points in the *i*th image plane; and ${\hat{p}}^{ij}$ are the re-projection of $P_{w}^{j}$ in the image plane. This problem can be solved using the Levenberg-Marquardt algorithm [19].

#### 2.2.3. External Parameters Self-Calibration

In a typical stereo-vision system, the cameras are fixed with respect to each other, and one of the cameras is chosen as the world coordinate system. If the stereo-vision system is vibrating during the measurement, it will move two camera coordinate systems and change the relative pose between the two cameras. To obtain accurate measurement results in a vibrating environment, the external parameters of two cameras need to be corrected in real time, and the world coordinate system has to be stationary during the measurement.

To gain an accurate solution of the relatively rigid motion R,t of the two cameras, the non-linear least-squares optimization method is used. According to Equation (5), the optimization cost function can be described by Equation (6):

(6) $$Cst={\sum_{i=1}^{M}\sum_{j=1}^{N}\left\|p_{1}^{ij}-{\hat{p}}_{1}^{ij}(A_{1},K_{1},R_{1}^{i},t_{1}^{i},P_{w}^{j})\right\|}^{2}+{\sum_{i=1}^{M}\sum_{j=1}^{N}\left\|p_{2}^{ij}-{\hat{p}}_{2}^{ij}(A_{2},K_{2},R_{2}^{i},t_{2}^{i},R,t,P_{w}^{j})\right\|}^{2}.$$

During iterative computation, the intrinsic camera matrixes, lens distortion parameters, and 3D coordinates of the reference points are fixed; only the external parameters of two cameras and the relative rigid motion are refined.

After the optimization, the translation vector is given in a metric reconstruction coordinate system, and it needs to be transformed into the Euclidean coordinate system using a scaling factor. If the distance between any two control points in the Euclidean coordinate system is known, it can be used to calculate the scaling factor. After calibrating the external parameters of both cameras, the 3D coordinates of target points can be calculated according to Equation (2). Note that the initial value of external parameters is important for the iterative computation, which may cause the iteration to not converge or fall into a local minimum. To solve this problem, an accurate non-iterative method [20] is used to calculate the initial value of external parameters.

If the world coordinate system is fixed on one of the cameras in the stereo-vision system, it will move in the vibration environment and the measurement error will increase. To solve this problem, the world coordinate system is defined by reference points that are stationary during the measuring process. During the forging process, the bracket of the forging machine can be considered to be stationary. Therefore, the reference points are placed on the bracket, which will be described in Section 3.2.

#### 2.2.4. Speed Solution

To compute the ram speed of a steam hammer, the measured points should be stable with respect to the steam hammer. Then, those measured points can move with the same speed and in the same direction as the steam hammer. Then, successive frame images of the stereo-vision system are used to compute the speed as follows:

(7) $$v=\frac{\Delta s}{\Delta t},$$

where v is the average speed of the measured point in time interval Δt, and Δs is the displacement of the measured point in time interval Δt.

It should be noted that, according to Equation (7), the speed measurement error is inversely proportional to the time interval. In actual application, the time interval is very short, only 0.25 ms, which will result in a large error in the speed measurement. In addition, if the displacement measurement error is smaller than the displacement Δs in time interval Δt, the measurement speed will be wrong. To solve this problem, the speed is calculated by calculating the derivative of the displacement curve, and the displacement curve is fitted by the least-squares method. If sufficient displacement data are measured, the speed can be accurately calculated.

## 3. Experiments

**Figure 3:**
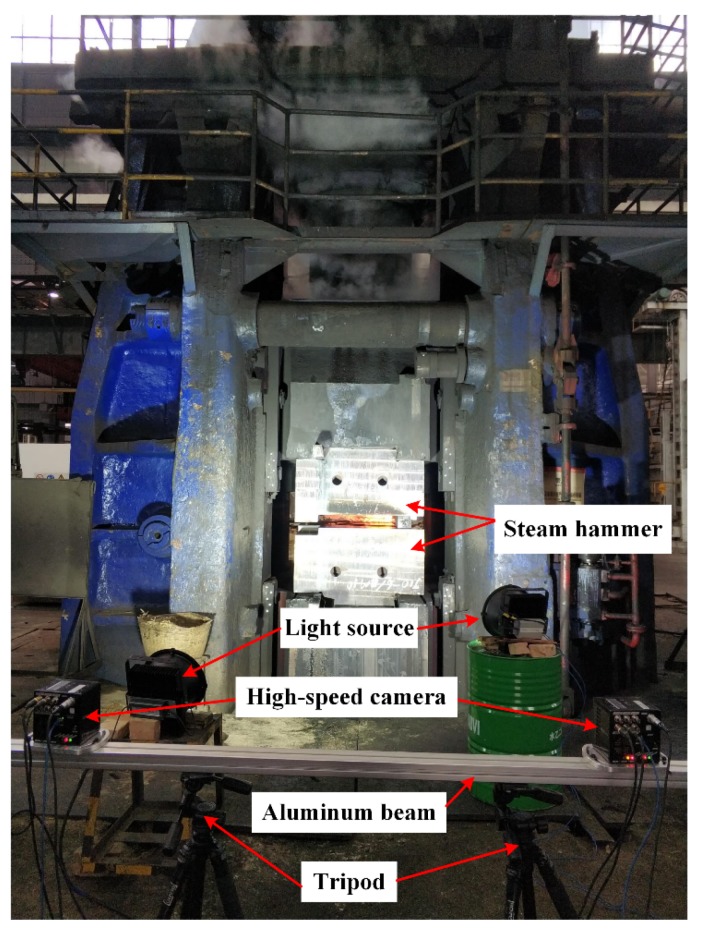
Experimental setup.

To verify the accuracy and effectiveness of the proposed method, we programmed it using Microsoft Visual Studio 2015 with C++ (Microsoft, Redmond, USA). The experimental arrangement is shown in Figure 3. The steam hammer in this experiment is a counterblow steam hammer in which each hammer has a weight of about 100 tons. In this section, the experiments are as follows: (1) Large FOV calibration experiment. A scale bar with known coded feature distance was measured 10 times while it was positioned in the measurement volume. This allowed the measured distance and reference values to be compared to evaluate the measurement accuracy. (2) External parameter calibration experiments. The 3D coordinates of reference points on the forging machine’s bracket were measured when the forging machine was operated, and the distances between them were analyzed to evaluate the dynamic measurement accuracy. (3) Ram speed measurement. The ram speed of the steam hammer was measured to verify the capability and effectiveness of the stereo-vision system.

### 3.1. Large FOV Calibration Experiment

**Figure 4:**
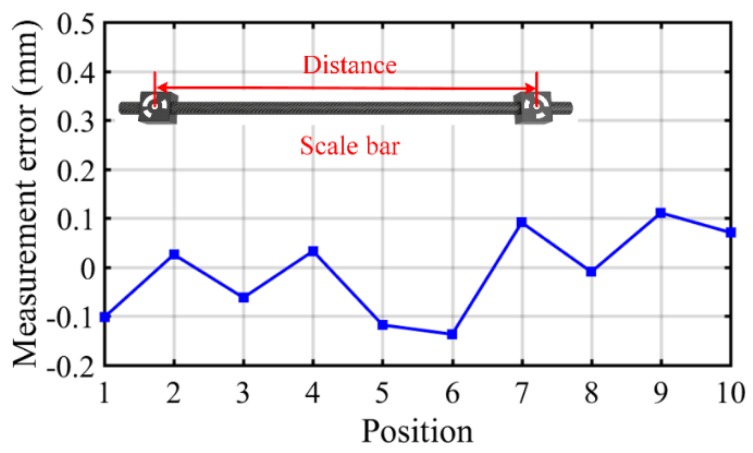
Measurement accuracy test results.

To test the measurement accuracy of the developed stereo-vision system, a scale bar with two ring-coded circle features was selected as the measured object according to the VDI-2634 Optical 3D measurement accuracy test standard. The distance between the centers of these two circles was accurately known. In the experiment, the internal parameters of the stereo-vision system were calibrated by placing the cross-shaped calibration target at 12 different orientations and positions within the measurement volume, then the external parameters could be calibrated using images of the calibration target. The scale bar was placed at 10 different orientations and positions within the measurement volume. The measurement range was approximately 3000 mm wide, 2400 mm tall, and 1000 mm deep, and the measurement distance was approximately 5400 mm. The measurement error is defined as the difference between the measured length of the ring-coded circle centers and the known value of 1001.762 mm. The results are shown in Figure 4. It can be seen that the measurement error of the stereo vision system was less than 0.15 mm, which verified that the stereo vision system can obtain accurate 3D results.

### 3.2. External Parameter Self-Calibration Experiments

**Figure 5:**
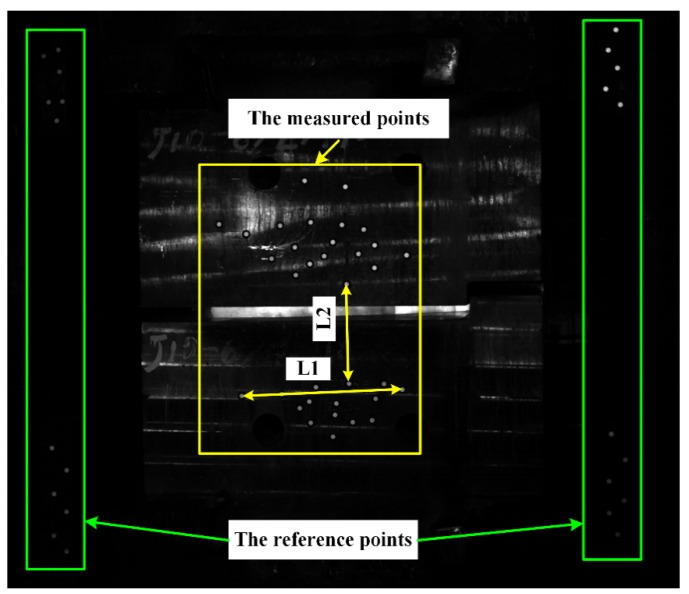
Reference points and measured points.

The stereo-vision system was vibrating while it measured the ram speed of the steam hammer. Therefore, it is necessary to evaluate the dynamic measurement accuracy of the stereo-vision system, which can be regarded as the difference of the measured distance between the measured points during the measurement. In other words, it is actually an analysis of the stability of measuring the distance between two measured points during the measurement. As shown in Figure 5, a number of retro-reflection targets on the forging machine’s bracket were chosen as reference points for synchronously calibrating the external parameters during the measurement. Before measurement, the 3D coordinates of all the reference points were accurately calibrated by the optical coordinate measuring system Creaform MaxSHOT 3D (Creaform, Lévis, Canada) [21], so the world coordinate system of the stereo-vision system is the measurement coordinate system of the optical coordinate measuring system. The retro-reflection targets on the steam hammer are the measured points.

**Figure 6:**
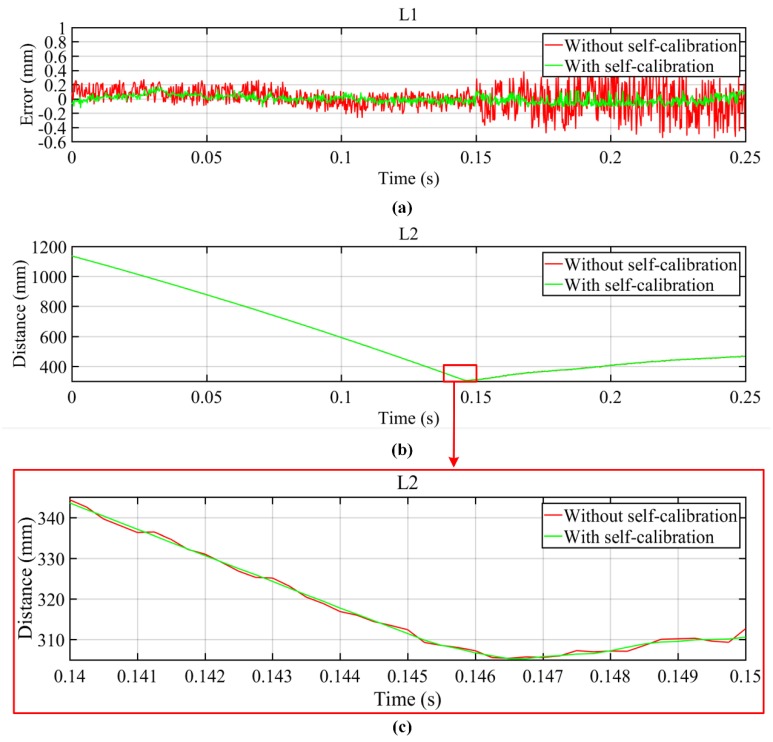
Dynamic measurement results for (**a**) distance error of L1, (**b**) distance of L2, and (**c**) distance of L2 at 0.14–0.15 s.

During the experiment, the upper and lower hammers moved downwards and upwards, respectively; 1000 images were captured by each camera with an acquisition speed of 4000 frames per second and exposure time of 0.000125 s. The measurement range was approximately 3000 mm wide, 2400 mm tall, and 1000 mm deep, and the measurement distance was approximately 5400 mm. As shown in Figure 5, two distances were measured within the measurement area. As the hammer moved, L2 changed, but L1 did not change. The error is defined as the difference between the measured distance of L1 and its known value. The measurement results are shown in Figure 6. Figure 6a shows that by using the self-calibration method, the measurement results were more accurate than without using the self-calibration method, and the measurement accuracy was less than 0.2 mm by using the self-calibration method. After the upper and lower steam hammers were in contact (where L2 reaches its minimum value), the measurement accuracy was significantly reduced when the self-calibration method was not used, as shown in Figure 6a,b. The reason for this is that when the upper and lower hammers were in contact, the vibration was at a maximum. This is shown in Figure 6c, where the distance curve is smoother when using the self-calibration than without using it. This also proves that the self-calibration method can effectively improve the measurement accuracy.

### 3.3. Ram Speed Measurement

**Figure 7:**
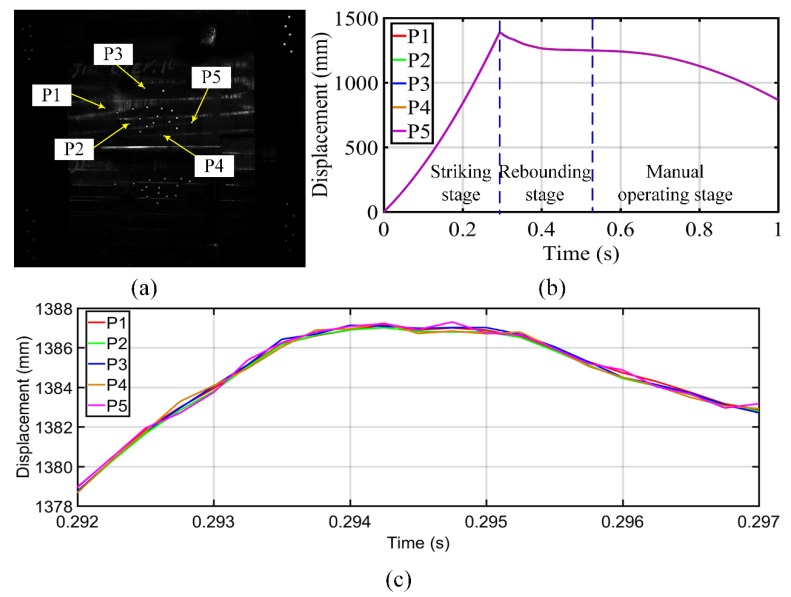
Displacement measurement for upper steam hammer: (**a**) measurement points and displacement of measured points at (**b**) 0–1 s and (**c**) 0.292–0.297 s.

In this experiment, a number of retro-reflection targets on the upper and lower hammers were measured to analyze their ram speeds, as shown in Figure 7a. The exposure time, measurement range, and measurement distance were the same as those described in Section 3.2. During the experiment, 4000 images were captured by each camera, but some scattered debris blocked the measurement points. Only five points on the upper hammer (shown in Figure 7a) could be measured during the whole process. The displacements of these points are shown in Figure 7b. It can be seen that the five displacement curves were almost the same because the upper hammer is a rigid body and the displacement of all points on the upper hammer should be the same at the same time. The displacement curves can also show the movement stage of the steam hammer as the displacement of points became larger during the striking stage. After the displacement reached the maximum, the steam hammer began to rebound and then rise under mechanical force, during which the displacement of these points became smaller.

**Figure 8:**
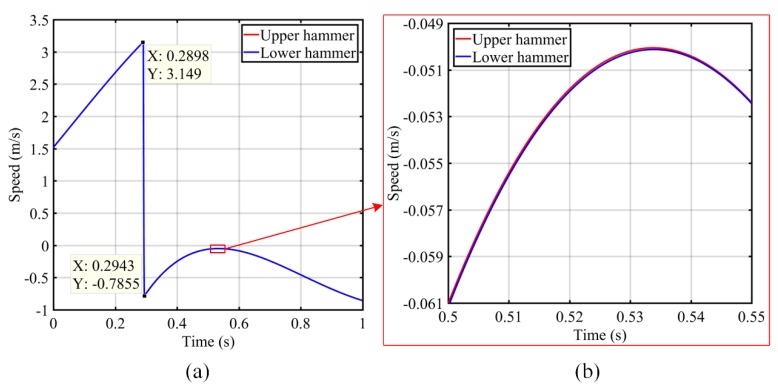
Ram speed of steam hammer at (**a**) 0–1 s and (**b**) 0.50–0.55 s.

Using this information, the speed can be calculated according to the speed solution described in Section 2.2. The speed of the upper and lower hammers is the average speed of the measured points on the upper and lower hammers separately, as shown in Figure 8. In the comparison of the speed of the steam hammer, the direction of the upper steam hammer’s velocity is downward and the direction of the lower steam hammer is upward. As shown in Figure 8a, the maximum speed of the upper and lower hammers is 3.149 m/s, and the rebounding speed is −0.7855 m/s. Figure 8b shows the speed history for 0.50–0.55 s, where the speeds of the upper and lower hammers were almost the same; this is consistent with the structure of the counterblow steam hammer. This proves that this method can achieve high accuracy in speed measurement.

## 4. Conclusions

This paper presents a stereo-vision system for measuring the ram speed of a steam hammer. To solve the problem of calibrating a stereo-vision system with a large FOV, a bundle-adjustment-principle-based method is proposed to realize high-accuracy calibration. This method can obtain accurate calibration results when the calibration target is not precisely manufactured. To decrease the influence of strong vibrations, a stationary world coordinate system was devised, and the external parameters were recalibrated during the entire measurement process. The accuracy and effectiveness of the proposed technique were verified by an experiment to measure the ram speed of counterblow steam hammers in a die forging device. In addition, this technique can be also used for displacement measurement in an environment with strong vibrations and a large FOV.
